# Photopatterned microswimmers with programmable motion without external stimuli

**DOI:** 10.1038/s41467-021-24996-8

**Published:** 2021-08-05

**Authors:** Yeongjae Choi, Cheolheon Park, Amos C. Lee, Junghyun Bae, Hyeli Kim, Hansol Choi, Seo woo Song, Yunjin Jeong, Jaewon Choi, Howon Lee, Sunghoon Kwon, Wook Park

**Affiliations:** 1 Nano Systems Institute, Seoul National University, Seoul, Republic of Korea; 2 Department of Electronic Engineering, Kyung Hee University, Gyeonggi-do, Republic of Korea; 3 Department of Electronics and Information Convergence Engineering, Institute for Wearable Convergence Electronics, Kyung Hee University, Gyeonggi-do, Republic of Korea; 4 Bio-MAX Institute, Seoul National University, Seoul, Republic of Korea; 5 Department of Electrical and Computer Engineering, Seoul National University, Seoul, Republic of Korea; 6 Integrated Major in Innovative Medical Science, Seoul National University, Seoul, Republic of Korea; 7 Interdisciplinary Program for Bioengineering, Seoul National University, Seoul, Republic of Korea; 8 Present Address: Wyss Institute for Biologically Inspired Engineering, Harvard University, Boston, MA USA

**Keywords:** Actuators, Gels and hydrogels, Design, synthesis and processing

## Abstract

We introduce highly programmable microscale swimmers driven by the Marangoni effect (Marangoni microswimmers) that can self-propel on the surface of water. Previous studies on Marangoni swimmers have shown the advantage of self-propulsion without external energy source or mechanical systems, by taking advantage of direct conversion from power source materials to mechanical energy. However, current developments on Marangoni microswimmers have limitations in their fabrication, thereby hindering their programmability and precise mass production. By introducing a photopatterning method, we generated Marangoni microswimmers with multiple functional parts with distinct material properties in high throughput. Furthermore, various motions such as time-dependent direction change and disassembly of swimmers without external stimuli are programmed into the Marangoni microswimmers.

## Abstract (web summary)

Maragoni microswimmers show the advantage of self-propulsion but their development is limited by fabrication techniques. Here, the authors use a photopatterning method which allows for a high throughput production of maragoni microswimmers with multiple functional parts and distinct materials properties.

## Introduction

In nature, over 1200 species of life have the ability to move on the surface of the water. While the majority of life physically stroke water when swimming, few creatures can swim by other principles. For example, Microvelia and Rove beetles can propel faster than the conventional physical swimming speed using the Marangoni effect^1^. Marangoni effect is a mass transfer phenomenon between two liquid regions with different surface tension^2,3^. These insects secrete surfactants that lower the surface tension of water and the Marangoni effect allows them to propel along the surface of the water from low to high surface tension areas. With an explosive speed of approximately twice the physical swimming speed, Microvelia uses the Marangoni effect to escape from predators^1,4^. Inspired by nature’s Marangoni effect-based swimming principles, various studies on robots on the surface of the water, seawater and organic solvent/water mixture were introduced^5–14^. Without the mechanical systems for stroking water or external stimuli, Marangoni swimmers demonstrate powerful swimming performance by producing mechanical energy directly from the surfactant^6,15^. With a simple fuel releasing system, recent studies have demonstrated that the Marangoni swimmer in milli- or microscale can swim tens of minutes in a linear or circular motion without an external energy source or control^11,12,16^. In contrast, the motion of conventional swimmers that are less than a few hundred micrometers in size generally requires external stimuli, such as lasers or ultrasound, as energy source^17–19^; this is because the smallest reported battery^20^ has an area of ~2 mm^2^ and there are substantial challenges in integrating such parts in microscale robotics. Furthermore, although external stimuli-based methods show a high level of programmability, the external stimuli sources are much bulkier than the actual swimmers, limiting the practical applications of such swimmers.

Although Marangoni microswimmers demonstrated powerful and autonomous swimming, there is a limitation in the precise mass fabrication, thereby affecting their scale, degree of programmability and future development. Fuels used for Marangoni swimmers are usually surfactants that exhibit amphiphilic properties, which causes them to dissolve after any liquid contact that is required during fabrication^6,10,16,21^. As a result, a variety of methods or techniques that include washing with solutions cannot be incorporated into the fabrication process of these swimmers. To program the motion of the swimmers, the fuel releasing component should be precisely positioned in the swimmers. Furthermore, multiple parts with a distinct function that control the motion of the swimmers should be integrated into these swimmers. Previously reported methods loaded liquid- or solid-phase fuels on pre-made swimmers by pipetting^22^ or hand assembly^12^ that have limitations in throughput, precision and scale to achieve mass fabrication of swimmers below millimeter scale, even though they achieved advanced functions such as prolonged motion or environmental sensing. Alternatively, a recent method reported machining of the fuel-immersed film in the designated conformation^16,22^. However, the described swimmers sporadically released their fuel to all sides without control, lowering their efficiency and programmability of motion. As a result, the current programmability of Marangoni microswimmers is limited.

In this study, a fuel for the highly programmable Marangoni microswimmers was introduced to overcome the fabrication problem. We introduced polyvinyl alcohol (PVA) as a fuel because it can be dissolved in water to lower the surface tension. PVA has poor solubility in organic solvents and is not dissolved when additional fabrication procedures based on non-polar solvents are performed^23,24^. As a result, we were able to introduce maskless photolithography^25–27^ to fabricate Marangoni microswimmers. Photolithography enables not only high-throughput and multi-scale fabrication but also iterated photolithography cycles, enabling the integration of multiple functional materials in a single swimmer. Using these methods, we introduced time-dependent programmable features for microswimmers, such as shape change and disassembly. These features influence the swimmers’ motion for a particular time-course as feedback. By utilizing these, we demonstrated time-dependent direction change or increment of the number of swimmers and its direction change, without external stimuli. Through the highly programmable properties of the proposed microswimmers, we demonstrate a proof-of-concept for their practical implementation in cargo delivery.

## Results

### Marangoni microswimmers with programmable motion

**Fig. 1:**
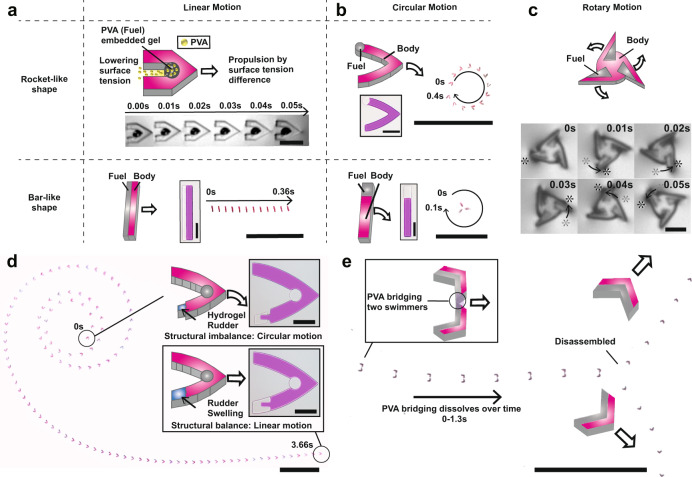
Schematic of Marangoni microswimmers with multifunctional parts for highly programmable motion. **a** PVA released from the fuel part lowers the surface tension of the adjacent areas and enables propulsion through the Marangoni effect. The hydrophobic body (red) enables swimmers to float on the surface of the solution. Middle (in black and white): FastCam images of a microswimmer with linear motion; scale bars represent 250 µm (black box and FastCam images) and 1 cm (zoom-out pictures). **b** Circular motion of swimmers enabled by selectively designing balance of the swimmer structure and surface tension profile; scale bars represent 250 µm (black boxes) and 1 cm (zoom-out pictures). **c** Rotary motion of the microswimmer. The asterisk marks the same wing of the microswimmer; the scale bar represents 100 μm. **d** Time-course motion change of the microswimmer. The size of the hydrogel rudder placed on the microswimmer changes due to swelling, which leads to a change in their motion from circular to linear. The volume ratio of PEGDA:Ethanol for the porous hydrogel rudder fabrication is 5:5; scale bar represents 250 µm (black boxes) and 1 cm (zoom-out pictures). **e** By bridging two swimmers with water-soluble PVA, swimmers are disassembled after a designated time. Scale bar represents 1 cm.

Figure 1 shows the concept of the proposed Marangoni microswimmer. The body of the swimmer is fabricated with polyurethane acrylate (PUA), allowing it to float on aqueous solutions owing to its hydrophobicity and density lower than 1. The fuel component is a PEG-based hydrogel containing solid-phase PVA. When the microswimmer is placed on water, the hydrogel absorbs the water and the PVA is dissolved (Fig. 1a). Previous studies report that the surface tension can be reduced by approximately 25% with 5% of PVA (average molecular weight of 70,000) in an aqueous solution than in pure water^23^. As a result, an unbalanced surface tension occurs around the microswimmer, causing it to move from low to high surface tension areas due to the Marangoni effect (Supplementary Movies 1 and 2). Moreover, since the hydrophobic body of the microswimmer guides the direction of the fuel release, a linear motion is observed. Swimmers can be propelled in different motions, depending on the shape and location of the body and the fuel (Fig. 1b, c). Previous studies on Marangoni microswimmers mainly demonstrated a simple shape-based propulsion direction in the absence of external stimuli. The directionality of Marangoni swimmers is determined by the propulsion force arising from the Marangoni effect and the hydrodynamic drag of the swimmers. For example, rocket-like swimmers or rectangular swimmers are reported to propel linearly because the propulsion and drag forces act in the same axis^6,11,16,22,28–32^. We demonstrate that these swimmers can also exhibit circular motion (Fig. 1b and Supplementary Movies 3, 4). If the fuel compartment is relocated from the center to the wing of a rocket-like swimmer, the area with the lowest surface tension shifts toward the wing. As a result, the propulsion force of the Marangoni effect shifts and the circular motion of the swimmer is achieved. In addition, even when the propulsion force (i.e., location of fuel compartment) is not shifted, drag force on the PUA-body shifts and changes its magnitude with respect to the length of the PUA body. Thus, we also achieved circular motion by adding such a body to a rectangular swimmer. Moreover, by increasing the size of the PUA-body part of the rectangular swimmers, we were able to control the curvature of circular motion (Supplementary Fig. 1 and Supplementary Note 1 for details). Therefore, the precise control of the fuel release and body shape also enables the motor-like motion of the microswimmers. In Fig. 1c and Supplementary Movie 5, we show that Marangoni swimmers can exhibit circular motion of up to 20 rotations per second. Furthermore, a parallel movement of multiple microswimmers with identical shapes was demonstrated (Supplementary Movie 6).

In this study, we were able to program the motions of microswimmers in a time course by programming different time-dependent variables. First, we demonstrate a Marangoni microswimmer that changes direction in time-course (Fig. 1d and Supplementary Movie 7). The rudder is a highly porous hydrogel designed to shrink and swell dramatically. As soon as it is fabricated and dried, the rudder shrinks to a minimal size. When the microswimmer touches the surface of the aqueous solution, a circular motion is observed due to the structural imbalance of the microswimmer. Then, the hydrogel rudder swells and the microswimmer gains a linear direction when its structure becomes balanced. The time of the change of direction can be determined through the characterization of the shrinking and swelling mechanics. In addition to the control of the propulsion direction via the rudder, we have demonstrated a time-dependent disassembly of a sub-microswimmer (Fig. 1e and Supplementary Movie 8). By bridging two microswimmers with PVA that dissolves during propulsion, we have shown that the two initially propel together before disassembling after a designated time. By designing different relative fuel positions before and after the disassembly, we were able to demonstrate the disassembly of the swimmers. To describe the time-course programmability of swimmers, we depicted diagrams for designing differently programmed motions (Supplementary Fig. 2). The type of programmable motion is determined by various parameters, namely: the amount of fuel, the balance of swimmers and the surface tension around the swimmers. These variables (rhombus) lead to different paths in the diagram, resulting in motion through the Marangoni effect (check functions in the rectangles).

### Fabrication process for Marangoni microswimmer

**Fig. 2:**
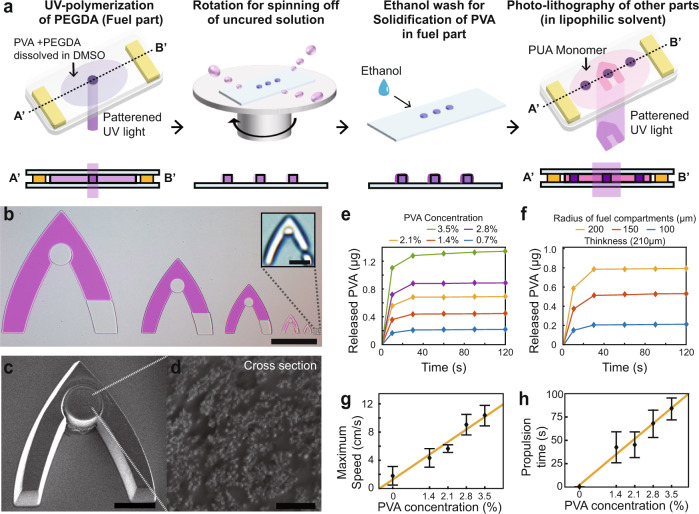
Photolithography of Marangoni microswimmers enables Marangoni microswimmers with multifunctional parts. **a** Fabrication method of microswimmers, including PVA-embedded gel; PVA dissolved in photocurable polymer solution does not react during UV polymerization. The uncured solution is spun off and ethanol is introduced to solidify the PVA in the polymeric mesh. An additional fabrication process using a hydrophobic solvent follows. **b** Variously sized microswimmers; scale bars represent 500 (down left) and 10 µm (upright). **c** Scanning electron microscope (SEM) image: single fuel component; scale bar represents 100 µm. **d** SEM image: fuel component cross-section; scale bar represents 3 µm. **e** Time-course profiles of PVA release from fuel compartment (disk of 200 µm radius, 210 µm thickness and 6.6 × 10^6^ µm^3^ volume) fabricated under various PVA concentration conditions. **f** Time-course profiles of PVA release from disks of various sizes. PVA concentration in pre-polymer solution is 0.7% and all disks have a thickness of 210 µm. **g** Maximum microswimmer speed versus PVA concentration added during fabrication, enabling the programmability of the propulsion time of microswimmers (*n* = 20). Rocket-like microswimmers with 750 μm axes were used. The error bars represent the standard deviation (s.d). **h** Microswimmer propulsion time versus PVA concentration added during the fabrication (*n* = 20). Rocket-like microswimmers with 750 μm axes were used. The error bars represent s.d.

Each part of the microswimmer was fabricated using irradiation-patterned UV on the monomer solution of a photocurable polymer (Fig. 2a, Supplementary Fig. 3). The proposed photolithography technique can produce structures sized from a few micrometers to millimeters^25–27^. The height of the microswimmers was determined by a spacer placed between glass slides. Consequently, the volume and the shape of the microswimmers were designed to pre-encode desired motions. To fabricate the fuel part, PVA was mixed with poly(ethylene glycol) diacrylate (PEGDA) 700 solution and the PEGDA was polymerized. Then, an ethanol wash was applied to replace the solvent in the pre-polymer solution. PVA aggregates and precipitates because ethanol does not dissolve PVA. To remove the residual monomer solution and eliminate the remaining PVA out of the fuel compartment, we spun off the uncured solution by rotating the substrate used for fabrication. Using this technique, we obtained a patterned fuel part. Previously, the conventional fabrication process could not be utilized for fabricating Marangoni microswimmers because the amphiphilic fuel was washed off in the wash process. However, by introducing hydrophilic PVA, the washing process using lipophilic agents was made possible. As a result, additional photolithographic processes in organic agents, such as ethanol, become possible. The adjacent compartment that was polymerized independently generated a crosslinked network with residual moieties of the previously fabricated compartment^33^, resulting in a swimmer with multiple functional materials. The UV exposure time was 0.1 s for one fuel component and one PUA body. In optimized conditions, 2400 microswimmers were fabricated per glass slide (*L* = 76.2 mm, *W* = 25.1 mm) in 12 min, including centrifugation and PVA solidification. By increasing the size of the glass slides, the number of microswimmers fabricated can be linearly increased. As a result of the fabrication process with programmable fuel loading, microswimmers with multiple parts on multiple scales were generated in a high-throughput manner (Fig. 2b). The PVA loaded through the proposed fabrication method was confirmed using a Scanning electron microscope (SEM) (Fig. 2c, d). When the cross-section of the hydrogel was observed through SEM (Fig. 2c), the structural difference between the PVA-loaded hydrogel and the hydrogel without PVA (Supplementary Fig. 4) was confirmed.

### Analysis of swimming performance considering the amount of fuel

By controlling the quantity of the fuel released through loading PVA in a scalable manner, we controlled the speed and the propulsion time (Fig. 2e–h). Fuel quantification was performed via the ultraviolet-visible (UV) spectrophotometry of the water with released PVA. The relationship between the PVA concentration in the water and the corresponding absorbance at the peak wavelength (*λ*_max_, 215 nm) was obtained as a reference (Supplementary Fig. 5). Subsequently, the absorbance of the solution in which the fuel compartment released loaded PVA was measured and the concentration of PVA was calculated based on the reference. The mass of PVA released was linearly proportional to the initial loading amount and size of the fuel (Fig. 2e, f and Supplementary Note 2). More than 90% of the fuel was released in 10 s and the release was completed within 2 min. Approximately 0.2–1.4 μg of PVA was released from the fuel compartment (disk of 200 µm diameter and 210 µm thickness) fabricated with 0.7–3.5% weight concentration of PVA (Fig. 2e). With the maximum concentration of PVA in the photolithography solution, rocket-like microswimmers with 750 μm axes were able to propel by more than 150 times the length of their axes within 1 s at their maximum speed (Fig. 2g, h and Supplementary Fig. 6, Supplementary Movie 9). Moreover, the microswimmers maintained motility for 80 s on average, while propelling by ~1.5 m. The maximum speed of the swimmers was achieved as soon as they touched the water surface. Then, the speed decreased, which is in accordance with the trend of PVA release from the fuel compartments. The maximum speed and propulsion time increased linearly, depending on the concentration of PVA in the pre-polymer solution (Supplementary Note 2 for a detailed discussion).

### Time-course motion change control by a hydrogel rudder

**Fig. 3:**
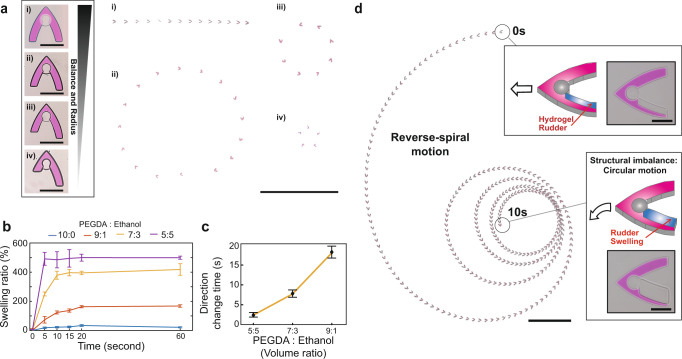
Hydrogel rudders enable time-dependent motion change of microswimmers. **a** By controlling the size difference between each wing of rocket-like swimmers, we were able to program swimmers motion from linear to circular with smaller diameters. In zoom-out pictures, the first circular motion of swimmers after it touches the water surface was displayed. Scale bar represents 500 μm (black boxes) and 1 cm (zoom-out pictures). **b** Swelling ratios of the hydrogel rudder (compared to the initial volume of fully dried gel) according to volume ratios of PEGDA to ethanol in the fabrication solution (*n* = 15). The swelling saturation time decreased as the ethanol ratio increased. The initial size of the hydrogel is 1.25 × 10^6^ µm^3^. **c** The time for motion change from circular to linear (the microswimmer in Fig. 1d) was programmed by changing the volume ratio of PEGDA to ethanol (*n* = 10). The size of the rudder is selected such that the swimmer is balanced when it fully swells. The error bars represent s.d. **d** Time-dependent motion change from linear to circular (reverse-spiral movement). Volume ratio of PEGDA:ethanol for the rudder fabrication was 9:1; scale bar represents 250 µm (black boxes) and 1 cm (zoom-out pictures).

By introducing rudder systems that change its volume dramatically according to time, swimmers could visit multiple unbalanced statuses and the radius their motion will change accordingly. The balance of the swimmers directly influences their motion by influencing the magnitude and direction of the hydrodynamic drag force (Fig. 3a and Supplementary Note 1). As the length difference of each wing of swimmers gets longer, swimmer’s motion is programmed from linear to circular motion with a smaller radius. As a result, circular to linear motion of the swimmers was achieved (Fig. 1e). The pre-polymer solution for the hydrogel rudder consisted of PEGDA and anhydrous ethyl alcohol. Anhydrous ethyl alcohol was used as a porogen to increase the pore size of the PEGDA hydrogel. To confirm the swelling ratio, we measured the swelling of hydrogels fabricated with PEGDA:anhydrous ethyl alcohol volume ratios ranging from 10:0 to 5:5 (Fig. 3b). The swelling speed increased with an increase in the ethyl alcohol ratio and the swelling ratio also increased upon increasing the volume of ethanol. In addition, the saturation time (time at which the hydrogel reaches 95% of its maximum volume) of the swelling decreased from ~20 to 5 s upon increasing the ethyl alcohol concentration. As the porosity increased, the diffusion constant and surface-to-volume ratio of the hydrogel increased. As a result, solution diffusion into the porous network was accelerated, the speed of swelling increased and the total time taken by the hydrogel to swell to its maximum volume decreased. In addition, for a constant scale and thickness, we observed that the size of the rudder was independent of the swelling ratio and speed if the polymer composition was identical (Supplementary Fig. 7 and Supplementary Note 2). Considering the swelling kinetics, we designed and fabricated a rudder with a specific size. The rudder could balance the swimmer and align the propulsion and drag forces as soon as it reached the swelling saturation volume. As a result, the time required for motion change from circular to linear increased from ~3 to 17 s as the PEGDA ratio increased (Fig. 3c). The time trend of the motion change agreed with the swelling saturation time. With the volume ratio of 9:1, microswimmers scanned through an area of ~12.57 cm^2^ with spiral propulsion, which is more than 25,000-fold larger than the area of the top face of a microswimmer (Supplementary Movie 10). In addition to the circular to linear-direction change, we also demonstrated linear to circular direction change by changing the rudder shape (Fig. 3d and Supplementary Movie 11). Compared with the previously demonstrated microswimmer propulsion that only followed the shape of its body for propulsion direction control, we introduce advanced microswimmers with time-course direction change within a designated period of time. Motion change microswimmers can be utilized for large-area applications that require numerous microswimmers in unstructured and narrow environments wherein the user cannot control individual microswimmers^15,34,35^. As a preliminary experiment, we tested the Marangoni microswimmers fabricated in this study for potential applications in environmental sensing (Supplementary Fig. 8). Owing to the hydrophobicity of the body, the microswimmers recognized oil and were assembled at the contaminated spot, propelling along the boundary between the pollutant and the water (Supplementary Fig. 8a, b). We released microswimmers programmed with a spiral trajectory on oil-polluted water. While moving in their spiral trajectory, the microswimmers scanned the environment and located the oil-polluted area. This preliminary result shows the potential of the proposed microswimmers to recognize and interact with environmental changes.

### Disassembly and motion change of microswimmers

**Fig. 4:**
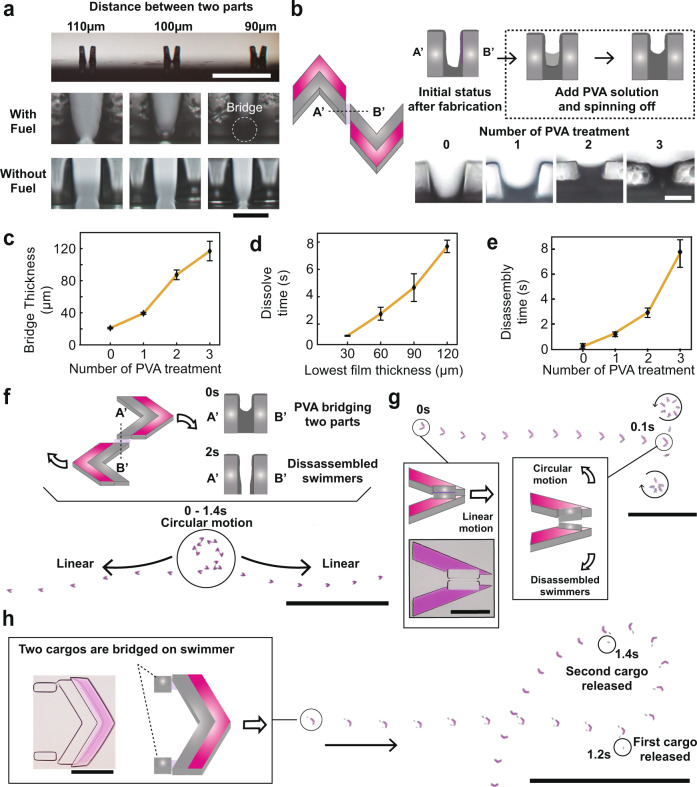
Disassembly and motion change of swimmers after a designated time. **a** By changing the distance between fuel parts to 100 µm, we were able to fabricate a water-soluble PVA bridge between these parts. 1 cm (white) and 100 µm (zoom-out pictures). **b**, **c** By controlling the thickness of the PVA bridge via the PVA layer stacking procedure, the thickness of the PVA bridge was increased (*n* = 10). Scale bars represent 100 µm. The error bars represent s.d. **d** As the thickness of the PVA film increased (Supplementary Fig. 9), the dissolution time needed to generate a 90 µm hole on the film increased (*n* = 10). The error bars represent s.d. **e** The disassembly time of the two rocket-like swimmers that were bridged was controlled based on the number of PVA treatments (*n* = 10). The error bars represent s.d. **f** By bridging the two linear-propelling swimmers moving in opposite directions, disassembly and a change in motion (from circular to linear) were demonstrated. Scale bar represents 500 µm (black boxes) and 1 cm (zoom-out pictures). **g** By bridging the bar-shaped swimmers in a rocket-shape, a change in motion from linear to circular was possible. Scale bar represents 500 µm (black boxes) and 1 cm (zoom-out pictures). **h** Cargo delivery concept. Two hydrogel cargos were bridged on single linear-propelling swimmers and released after the designated time. Scale bars represent 500 µm (zoom-in picture) and 1 cm (zoom-out picture). For microswimmers used for the **c**, **e**, one layer of PVA stacking was applied.

Inspired by the spinning-off wash step of the proposed fabrication of fuel parts, we introduced disassembly of swimmers during the propulsion (Fig. 4). We found that part of the uncured polymer resin is not washed and remains between the parts that shorter than 100 µm (Fig. 4a). Since we design enough space between the other demonstrations’ fuel parts, the uncured polymer resin does not affect swimmers’ performance. However, if we fabricate fuel parts of two independent swimmers closer than 100 µm, the parts are bridged by solidified PVA after fabrication. If these bridged swimmers are placed on the water, it propels together and disassembles as soon as the PVA bridge dissolves. Using this, we were able to program different propulsion motions of swimmers before and after disassembly, by changing the structure of the body and the fuel parts. We demonstrated disassembly and direction change of the swimmers from linear to circular (Fig. 1e and Supplementary Movie 7). To increase controllability of disassembly, we stacked additional PVA layers on the bridge to control the bridge’s thickness and the microswimmers’ disassembly time accordingly (Fig. 4b). The layers of PVA are introduced by adding PVA solution to the bridge and solidifying it. The PVA solution on the bridge can be added by pouring the PVA solution on the swimmer and spinning it off to retain solutions only on the indented area on the bridge. Subsequently, we treated the PVA precipitate with ethanol. We observed that the bridge thickness increased with the number of treatments. To quantify the kinetics of disassembly, we created a model for measuring the disassembly independently from the propulsion of the swimmers. To reproduce the dissolution of the bridge, we first fabricated films with various thicknesses. Specifically, PVA solutions were poured on glass with a spacer and ethanol was added to precipitate the PVA (Supplementary Fig. 9). We applied water and measured the time for the lowest part of the film to be dissolved and generate a hole with a 90 µm diameter (Fig. 4c). We chose a diameter of 90 µm because the distance between the two compartments that were bridged was 90 µm in the experiments. We determined that as the thickness of the film increased, the dissolution time increased linearly (Fig. 4d). Based on this model, we were able to program the disassembly times of the two rocket-like swimmers (Fig. 4e). Following changes in the bridge thickness according to the PVA treatment, the disassembly time increased and the trend matched that of the film model.

As a result, we were able to control swimmers’ disassembly time, from less than one second to around ten seconds. Utilizing the controlled disassembly of swimmers, we were able to demonstrate multiple scenarios of disassembly in programmed time (Fig. 4f–h). By bridging two rocket-like swimmers in the opposite or in the same direction, we were able to demonstrate disassembly and a change in motion (from circular to linear) or disassembly while maintaining motion (linear) (Fig. 4f, g and Supplementary Movies 12, 13). Other than bridging swimmers, we were able to bridge cargo-like hydrogels on one swimmer. At a designated time, the swimmer could release the cargos and escape the target area (Fig. 4h).

## Discussion

We developed a Marangoni microswimmer with multiple parts and integrated time-course programmed motions into the swimmers. Previously reported swimmers were limited in the throughput and precision of their fabrication and, as a result, the programmability of their motion was limited. In this article, we overcame these limitations by introducing PVA as fuel and also enabling the fabrication of microswimmers with multiple parts, integrated with a centrifugation-based wash method. With advances in robust and complex fabrication, we believe that our platform will accelerate the study of the kinetics of Marangoni swimmers and future research on higher programmability and various applications. To our knowledge, the overall kinetics of Marangoni swimmers have remained scarcely understood. With the various models provided herein, kinetics theories can be validated through experiments with multiple variants in terms of shape, fuel concentration and scale. Regarding applicability, PVA is bio-degradable and eco-friendly, so it will maximize the utility of microswimmers in a variety of environments in future. Introduced swimmers are applicable to swim on water-based liquid such as seawater or blood, since PVA can dissolves and lower the surface tension. With various motions and disassembly without any stimuli after releasing, microswimmers will be applicable in microenvironments or microchannel networks where only the inlet is exposed and changes dynamically. Moreover, the disassembly concept of swimmers also could be applied to cargo delivery. Also, studying the collective motions of multiple swimmers will be another area of study. When a large number of microswimmers moves in a limited space, the propulsion of the swimmer will be limited due to lowered surface tension of water after the release of the fuel. To overcome the problem, a fuel that changes its surface tension after a designated time is required.

## Methods

### Materials

To fabricate microswimmers functionalized for swimming, we used a 6.5:3.5 volume ratio of poly(ethylene glycol) diacrylate (PEGDA) (Aldrich) mixed with PVA (87–90% hydrolyzed, average mol wt 30,000–70,000, Aldrich) dissolved in dimethyl sulfoxide (Aldrich) as the fuel component or polyurethane acrylate (PUA) (minuta) as the body compartment. To fabricate the rudder, we used a mix of PEGDA and ethyl alcohol (99%, Daejung) in indicated ratio. 10 vol% of Irgacure 1173 (BASF) to the mixed solution as the photoinitiator. To trace swimming, 0.25 wt% of methacryloxyethyl thiocarbamoyl rhodamine B (Polysciences) was added to the PUA. To solidify the PVA, anhydrous ethyl was used as a wash solution. To fabricate microswimmers with multiple compartments, 3-(trimethoxysilyl)propyl methacrylate (TMSPMA) (Aldrich)-coated glass slides were used to create compartments on the glass sides.

### Maskless photolithography

The pre-polymer resin was dispensed between the polydimethylsiloxane-coated glass slides and the TMSPMA-coated glass slides with proper spacer materials that determined the thickness of the microswimmer. Microswimmers of various sizes were photopolymerized by modulating UV light (Lightningcure LC8, Hg-Xe lamp, Hamamatsh) using a digital micromirror device (DMD) (Texas Instruments). An optical microscope (IX71, Olympus) was aligned with the UV light source and the DMD and the patterned UV light was projected on the prepared polymer resin with a ×10 (NA 0.3), exposure power of 20 mW/cm^2^. For fuel parts, spin-coating treatment is proceeded for 90 s (1500 rpm 30 s, 3000 rpm 60 s) to remove the uncured resin.

### Swimming test

After fabrication, the microswimmers were detached from the TMSPMA-coated glass slide by gentle scraping. Then, the microswimmers were selected and released on the water surface using a tweezer. The motion of the microswimmers was filmed using a smartphone (iPhone 8, Apple Inc.) or a FastCam (MC2.1, Photron) and analyzed using ImageJ. Specifically, each movie frame was extracted and then the microswimmers were detected in contrast to the background. Further, the information obtained for the microswimmers’ location was used for speed calculation. The propulsion time of the swimmers was measured from the start of the experiment to the time when the speed of the swimmers reached the average speed of swimmers without fuel (~0.3 cm/s, which may occur because of external vibrations, wind, or Brownian motion). Regarding disassembly, the distance between the two bridged compartments was 90 µm in the entire experiment using the PVA bridge.

### UV–Vis spectra

The absorbance of PVA from the fuel compartment, dissolved in distilled water, was measured using a NanoDrop™ 2000/2000c spectrophotometer to detect the low wavelength (200 nm) of PVA. Further, it is difficult to measure the released PVA concentration of a single drug compartment owing to its low quantity; consequently, multiple compartments (224 per ml) were incubated and the absorbance was measured subsequently.

## Supplementary information

Supplementary Information

Description of Additional Supplementary Files

Supplementary Movie 1

Supplementary Movie 2

Supplementary Movie 3

Supplementary Movie 4

Supplementary Movie 5

Supplementary Movie 6

Supplementary Movie 7

Supplementary Movie 8

Supplementary Movie 9

Supplementary Movie 10

Supplementary Movie 11

Supplementary Movie 12

Supplementary Movie 13

## Data Availability

All data generated or analyzed during this study are included in the published article and its Supplementary Information and are available from the corresponding author on reasonable request.

## Code availability

This study did not use custom code that is deemed central to the conclusions.
