# Supplementary material for: Photopatterned microswimmers with programmable motion without external stimuli
Source: Nat Commun. 2021 Aug 5;12:4724. doi: 10.1038/s41467-021-24996-8 (PMC8342497; doi:10.1038/s41467-021-24996-8)
Supplement: Supplementary file 1 — Supplementary Information [file 41467_2021_24996_MOESM1_ESM.pdf]

Supplementary Materials for

Stimuli Independent Time Course Motion Change and Disassembly of Photopatterned Marangoni Microswimmers

Correspondence should be addressed to

Y.C. (yeongjae@snu.ac.kr), S.K. (skwon@snu.ac.kr), or W.P. (parkwook@khu.ac.kr)

This file includes:

Supplementary Note 1, 2

Supplementary Figure 1 to 9

Supplementary Note 1 – Kinetics Model of Marangoni Microswimmers

Based on prior research[1,2], we determined the relationship between fuel concentration, size, and speed. As approximated in the previous research[1], Re is not small (i.e., Re>>1) for swimmers larger than 300 μm, which is similar to our model. Accordingly, the propulsion force and dragging force can be approximated as follows:

$$F_{prop} \approx \Delta\gamma \, l_{contact}$$

Δγ is the asymmetric surface tension gradient, and $l_{contact}$ is the motor body contact line.

$$F_{drag} = \frac{1}{2}\rho v^2 C_d A$$

where ρ is the fluid density, v is the velocity, Cd is the drag coefficient, and A is the area.

Rectangular swimmers were previously reported[3,4] to propel linearly perpendicular to their longest edge, which has a lower surface tension than the shorter edge because of the larger surface area that releases the surfactant. However, although multiple studies have applied rectangular swimmers as a model system, directionality is observed to be random, with a fixed axis (perpendicular to the longest edge)[3,4]. Ideally, a swimmer would not exhibit movement along this axis when considering the shape symmetry and balanced surface tension along the propelling axis. Regarding this, a recent work[5] speculates that the spatial distribution of pores in a fuel compartment that releases fuel could give rise to small anisotropies in surface tension and initiate motion. In summary, if only the fuel part is present without the body part in Fig. 1b, the swimmer will be propelled linearly perpendicular to its longest edge, and the directionality will be random, with a fixed axis. In accordance with this, in our system, we observed that the directionality was clockwise or counter-clockwise in a circle for bar-shaped swimmers with the fuel and body parts. This randomness lowers the controllability and may affect the future applications of microswimmers. We point out that this problem can be mitigated by designing the body shape to control the release direction of the fuel (Fig. 1c).

In our study, with advances in the fabrication technique, we were able to control the propulsion direction of bar-shaped microswimmers. In the case of the bar-shaped swimmer in Fig 1b, the direction and magnitude of the drag force on the PUA body change with the length of the

PUA body, without any changes the propulsion force (i.e., fuel). As a result, as the length of the body increases, the curvature of the circular motion increases. We observed that swimmers with longer bodies exhibited higher curvatures, as expected (Supplementary Fig.1).

In addition, from the above approximation, we can calculate the maximum velocity v_{max} in the transient state (F_{prop}-F_{drag} = 0):

$$v_{max} = \sqrt{\frac{2\Delta\gamma \, l_{contact}}{\rho C_d A}}$$

Assuming that the swimmer has the same geometry and size, v_{max} will depend on the asymmetric surface tension gradient. Here, the surface tension of the water is dramatically lowered by the low quantity of PVA released[6], which is similar to the concentration used in the fabrication. As a result, v_{max} depended on the PVA concentration. In addition, we can expect that when the size of the swimmer is increased for a given amount of fuel released, the maximum speed will decrease in accordance with the inverse square root function. When considering that 90% of the PVA release is completed before the first 10 s of the swimmer's propulsion, the drag force, which increases in proportion to v^2, will be the dominant force. As a result, we observed an exponential decay of the speed.

Supplementary Note 2 – Size effect on fuel release and swelling

Depending on the size of the fuel compartment, the total PVA increased linearly according to its volume (Fig. 2d). However, the proportion of the fuel released from the fuel compartment did not change with the fuel compartment size (i.e., rapid release for 10 s; the remaining amount was released in 2 min). In addition, for the same scale and thickness, we observed that the size of the rudder was independent of the swelling ratio and speed if the polymer composition was identical (Supplementary Fig.7). For micro-scale hydrogels, owing to the high surface-to-volume ratio and water absorption rate of the microscale PEGDA gel, the effect of size was minimized. Furthermore, the effect of the size of the fuel part is governed by the diffusion time of the solution into the polymer mesh, which is given by[7]

$$Time = \frac{r^2}{D_{gel}}$$

where D_{gel} is the diffusion coefficient and r is the length traveled by the solution. Here, even if the size of the proposed swimmer increases, its height (thickness) is fixed, and only the area of the horizontal portion increases. Therefore, r is fixed, and all swimmers should have the same solvent diffusion time. In summary, as long as we fix the thickness of the swimmer and its scale (millimeters or micrometers), the size effect on the fuel release (proportion of PVA released per unit time) and swelling ratio will be negligible. Depending on the size (e.g., in the case of centimeter-scale hydrogel), we believe that the PVA release profile and swelling profile may change owing to the change in penetration of the solutions into the gel from the surrounding environment.

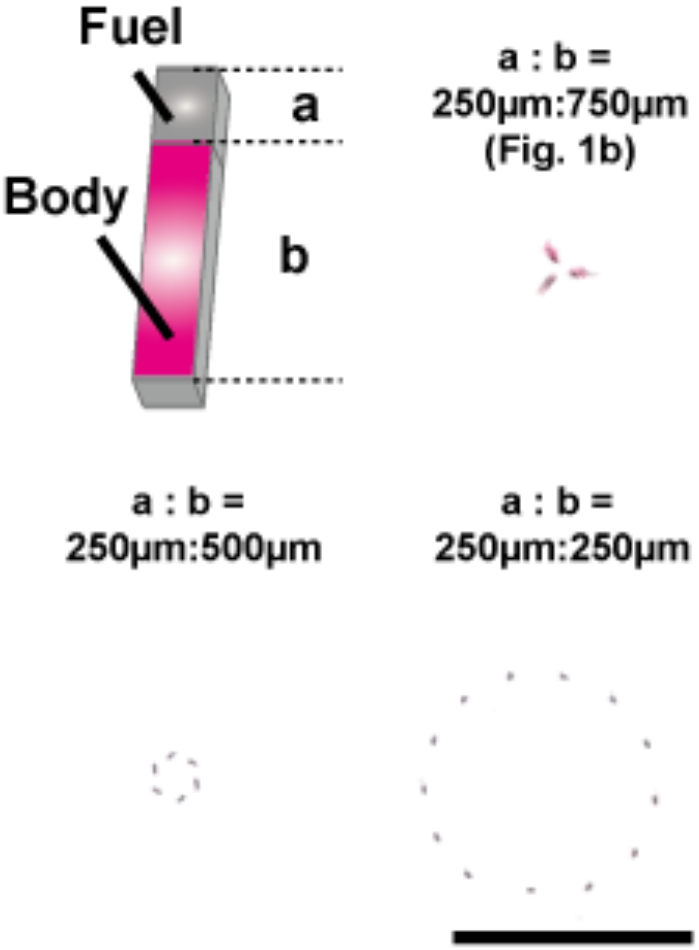

Supplementary Figure 1. By changing the ratio between fuel and body parts, circular motions with various curvatures are achieved via changes in the drag force applied to the swimmers. The first circular trajectory of a swimmer after it touches the water surface is displayed. Scale bar represents 1 cm (zoomed-out pictures).

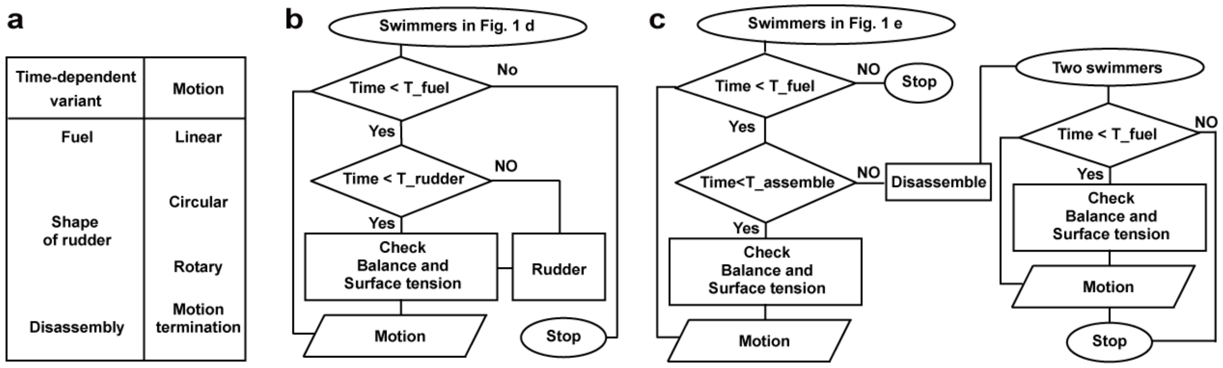

Supplementary Figure 2. a-c, Flow chart of encoded motion corresponding to the swimmers illustrated in Fig. 1 d,e.

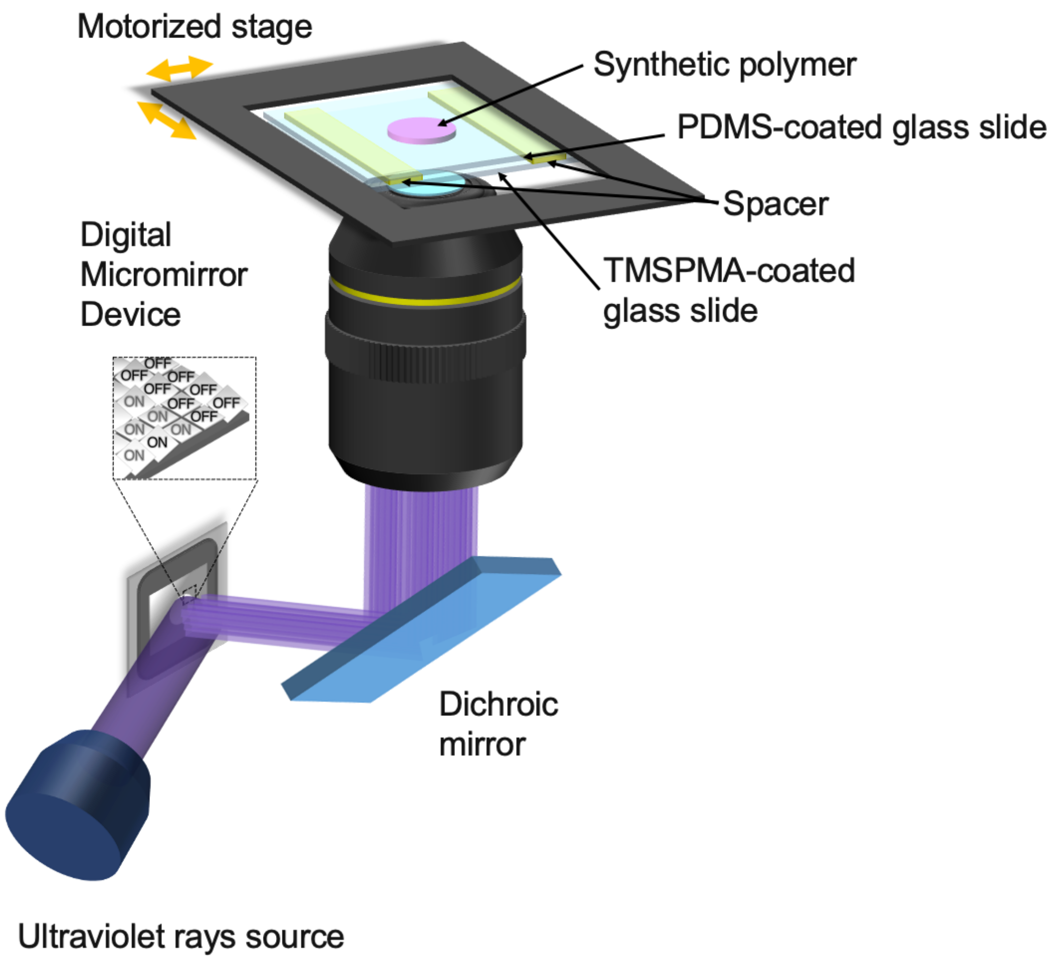

Supplementary Figure 3. Illustration of the optofluidic lithography setup.

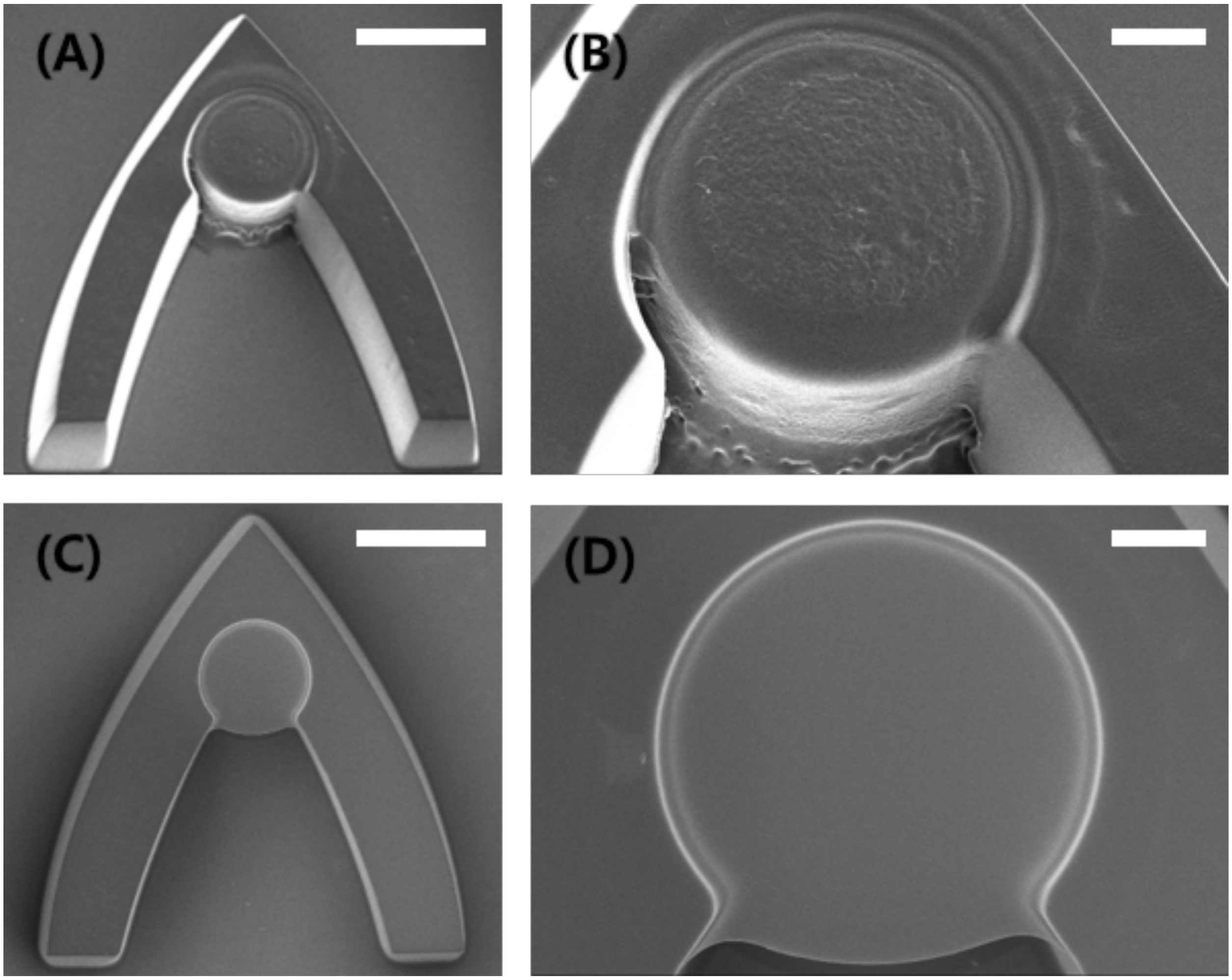

Supplementary Figure 4. SEM images of Marangoni microswimmer on the TMSPMA-coated glass slide. a Tilted SEM image of the Marangoni swimmer. **b** A magnified image of fuel component. The porous surface is confirmed by evaporation of PVA. **c** SEM image of the Marangoni swimmer without PVA in fuel component. **d** A magnified image of fuel component. The flat surface is confirmed due to non-PVA. (Scale bar: 100, 50, 100, 50 μm)

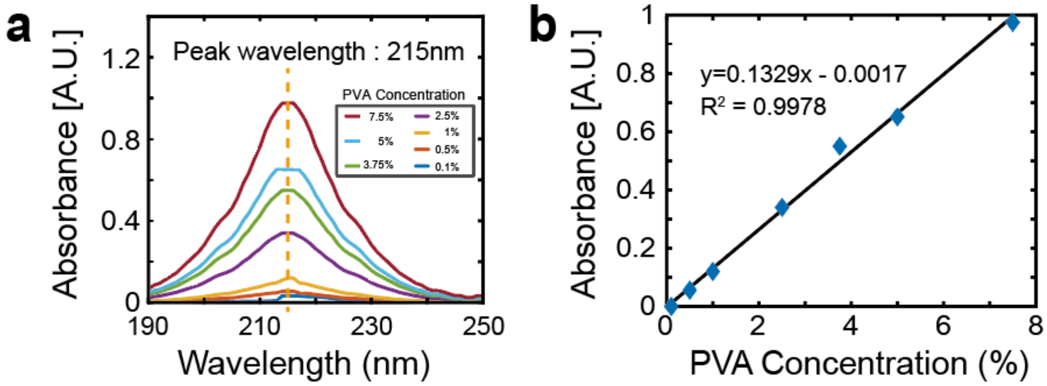

Supplementary Figure 5. **a** UV-Vis absorbance of reference solution. **b** Reference curve of absorbance–concentration relationship of PVA. Absorbance at the wavelength of 215 nm (λ_{max}) was used for deriving the curve.

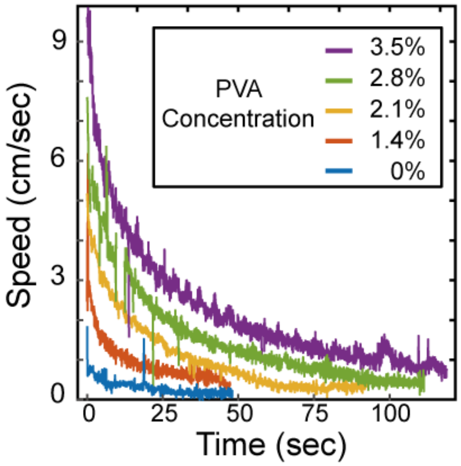

Supplementary Figure 6. Speed of microswimmer according to PVA concentration of pre-polymer solution.

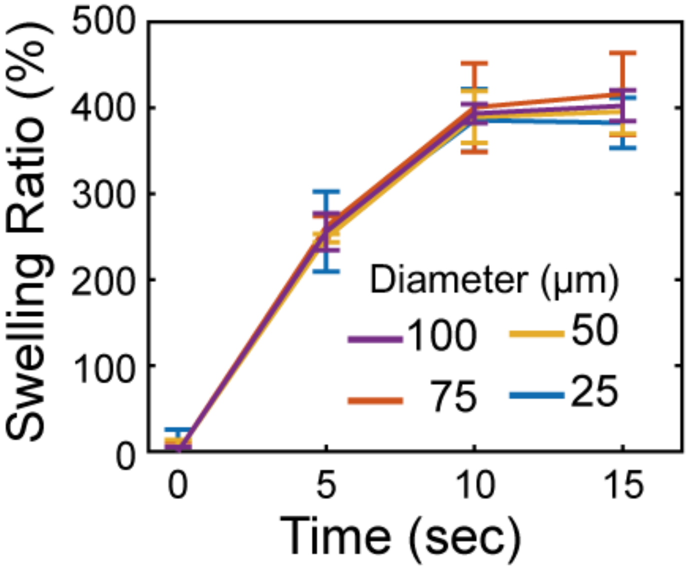

Supplementary Figure 7. Swelling ratio of the disk-like hydrogel with 75 μm thickness (fully dried) with respect to diameter (n=10). The volume ratio of PEGDA to ethanol in the fabrication solution is 7:3.

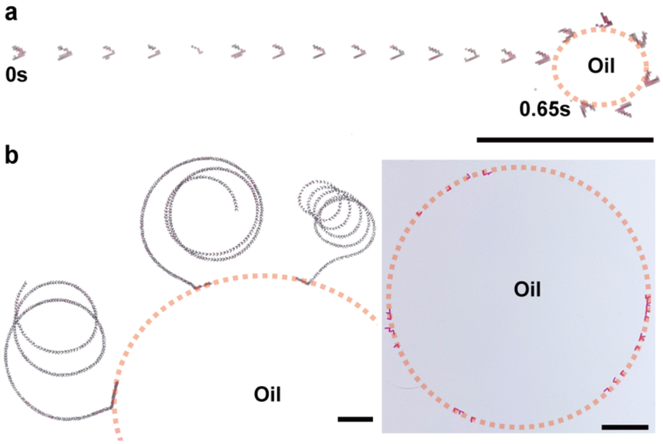

Supplementary Figure 8. Future application of proposed microswimmers. a Schematic of oil contaminant recognition using microswimmers. As soon as the microswimmers come into contact with oil, they are accumulated at the contaminated spot owing to the hydrophobicity of their bodies. **b** Microswimmers exhibiting spiral movement scanned an oil-polluted area and sensed the contaminated spot; Orange: pseudo-color of the oil area; scale bar represents 1 cm. To simulate an oil-polluted environment, an oil droplet (~1 mL; Mineral Oil, Sigma) was dropped in a water-filled petri dish (ϕ = 90 mm or 180 mm).

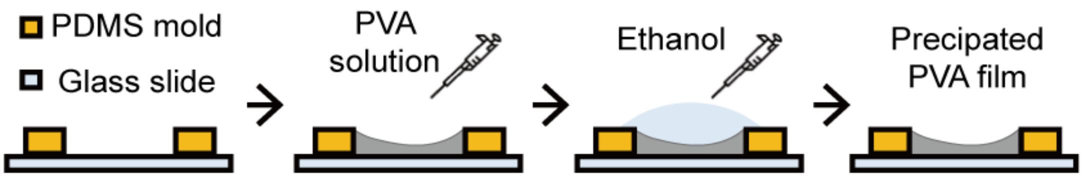

Supplementary Figure 9. Fabrication process of PVA film and the corresponding dissolution experiment.

References:

1. Pena-Francesch, A., Giltinan, J., & Sitti, M. Multifunctional and biodegradable self-propelled protein motors. Nat. Commun. **10**, 3188 (2019).
2. Zhang, H., Duan, W., Liu, L., & Sen, A. Depolymerization-powered autonomous motors using biocompatible fuel. *J. Am. Chem. Soc.* **135**, 15734–15737 (2013).
3. Park, J. H., Lach, S., Polev, K., Granick, S., & Grzybowski, B. A. Metal-organic framework 'swimmers' with energy-efficient autonomous motility. *ACS Nano* **11**, 10914–10923 (2017).
4. Bassik, N., Abebe, B. T., & Gracias, D. H. Solvent driven motion of lithographically fabricated gels. *Langmuir* **24**, 12158–12163 (2008).
5. Ender, H., Froin, A. K., Rehage, H., & Kierfeld, J. Surfactant-loaded capsules as Marangoni microswimmers at the air–water interface: Symmetry breaking and spontaneous propulsion by surfactant diffusion and advection. *Eur. Phys. J. E* **44**, 1–22 (2021).
6. Bhattacharya, A. & Ray, P. Studies on surface tension of poly (vinyl alcohol): Effect of concentration, temperature, and addition of chaotropic agents. *J. Appl. Polym. Sci.* **93**, 122–130 (2004).
7. Van Der Linden, H. J., Herber, S., Olthuis, W., & Bergveld, P. Stimulus-sensitive hydrogels and their applications in chemical (micro) analysis. *Analyst* **128**, 325–331 (2003).
