# Supplementary material for: Photopatterned microswimmers with programmable motion without external stimuli
Source: Nat Commun. 2021 Aug 5;12:4724. doi: 10.1038/s41467-021-24996-8 (PMC8342497; doi:10.1038/s41467-021-24996-8)
Supplement: Supplementary file 2 — Description of Additional Supplementary Files [file 41467_2021_24996_MOESM2_ESM.pdf]

Description of Additional Supplementary Files

File Name: Supplementary Movie 1
Description: Linear propulsion of the rocket-like swimmer.

File Name: Supplementary Movie 2
Description: Linear propulsion of the rectangular swimmer.

File Name: Supplementary Movie 3
Description: Circular motion of the rectangular swimmer.

File Name: Supplementary Movie 4
Description: Circular motion of the rocket-like swimmer.

File Name: Supplementary Movie 5
Description: Rotary motion of the microswimmer.

File Name: Supplementary Movie 6
Description: A parallel movement of multiple microswimmers with identical shapes.

File Name: Supplementary Movie 7
Description: Time-course motion change of the microswimmer.

File Name: Supplementary Movie 8
Description: Disassembly of the microswimmer after a designated time.

File Name: Supplementary Movie 9
Description: Long-range (1.5m) propulsion of the rocket-like microswimmer

File Name: Supplementary Movie 10
Description: Spiral movement of the microswimmer.

File Name: Supplementary Movie 11
Description: Reverse-spiral movement of the microswimmer.

File Name: Supplementary Movie 12
Description: Disassembly and a change in motion (from circular to linear) of the microswimmer.

File Name: Supplementary Movie 13
Description: Disassembly and a change in motion (from linear to circular) of the microswimmer
